# Post-Graduate Study in the Rotunda Hospital

**Published:** 1910-04-16

**Authors:** E. Hastings Tweedy

**Affiliations:** Master Rotunda Hospital. Rotunda Hospital, Dublin


*To the Editor of* The Hospital.

### POST-GRADUATE STUDY IN THE ROTUNDA HOSPITAL.

*To the Editor of* The Hospital.

Sir,—In this week's number of The Hospital, p. 45, it is stated in respect to post-graduate work in the Rotunda Hospital that "Matriculates enter as 'volunteer physicians' for a period of three months, for which they have to pay a fee of 30 guineas. In spite of the high fee, they have also to provide their own meals."

This information is entirely inaccurate and should not have been written. "Volunteer physicians" can enter for periods of from one to six months and are not confined to a three-months' course.

The fee charged for a three-months' course is 12 guineas (£12 12s.), not 30 guineas as stated.

It is not incumbent on the "volunteer physicians" to reside in the hospital, and many prefer to stop at some one of the numerous private hotels in its neighbourhood. Accommodation is provided in the hospital for those who desire to live in it, and they are charged 25s. per week for full board and lodging.

I remain, yours faithfully,

Rotunda Hospital, Dublin, April 10. E. Hastings Tweedy, Master Rotunda Hospital.

[We are glad to correct the statement with regard to the fees charged. The second part of the statement, that graduate students provide their own meals, is borne out by Dr. Tweedy's letter.—Ed. The Hospital.]

